# IMPACT OF ART-BASED INTERVENTION ON BRAIN FUNCTIONAL CONNECTIVITY IN OLDER ADULTS ALONG THE ALZHEIMER’S CONTINUUM

**DOI:** 10.1093/geroni/igae098.3032

**Published:** 2024-12-31

**Authors:** Chenshan Huang, Yuanjiao Yan, Yanhong Shi, Rong Lin, Hong Li

**Affiliations:** Fujian Medical University, Fuzhou, Fujian, China (People’s Republic); Fujian Provincial Hospital, Fuzhou, Fujian, China (People’s Republic); Fujian Medical University, Fuzhou, Fujian, China (People’s Republic); Fujian Medical University, Fuzhou, Fujian, China (People’s Republic); Fujian Medical University, Fuzhou, Fujian, China (People’s Republic)

## Abstract

**Background:**

Combined art-based interventions (CAIs) are considered effective treatment options for older adults along the Alzheimer’s disease (AD) continuum; however, the neural mechanisms underlying associated changes in neurocognitive performance remain unclear. Thus, we aimed to investigate the impact of a CAI programme in older adults along the AD continuum and to understand its mechanism.

**Methods:**

This parallel-arm randomised controlled trial was conducted between April 2021 and January 2023. Participants were randomised in a 1:1 ratio to either intervention group (IG) or waitlist control group (WG). The IG underwent a 16-week CAI programme. Neuropsychological assessments and magnetic resonance imaging were conducted before and after the intervention.

**Results:**

After the intervention, the IG showed greater improvement in general cognitive function, language, and memory than the WG. Significant differences were observed in the functional connectivity (FC) values in the temporal and cerebellar anterior lobes, fusiform, inferior occipital, and lingual gyri, and perirhinal and visual cortices between the groups. Further analyses showed that FC values reduced in these regions in the IG. In addition, changes in FC values were positively correlated with those in neuropsychological test scores in the IG.

**Conclusions:**

Our study suggests that the CAI programme can effectively improve general cognitive function, language, and memory in older adults along the AD continuum. These improvements may be changed due to decreases in FC in key brain regions, deepening the understanding of the neurocentral mechanisms that act as a tool for improving cognitive function.

